# The diel vertical distribution and carbon biomass of the zooplankton community in the Caroline Seamount area of the western tropical Pacific Ocean

**DOI:** 10.1038/s41598-022-23522-0

**Published:** 2022-11-07

**Authors:** Zhencheng Tao, Haochen Xian, Zhendong Luan, Feng Nan, Yanqing Wang, Song Sun

**Affiliations:** 1 Key Laboratory of Marine Ecology and Environmental Sciences, Institute of Oceanology, Chinese Academy of Sciences, Qingdao, 266071 China; 2 Laboratory for Marine Ecology and Environmental Science, Qingdao National Laboratory for Marine Science and Technology, Qingdao, 266237 China; 3 Center for Ocean Mega-Science, Chinese Academy of Sciences, Qingdao, 266071 China; 4 University of Chinese Academy of Sciences, Beijing, 100049 China; 5 Key Laboratory of Marine Geology and Environment, Institute of Oceanology, Chinese Academy of Sciences, Qingdao, 266071 China; 6 Key Laboratory of Ocean Circulation and Wave Studies, Institute of Oceanology, Chinese Academy of Sciences, Qingdao, 266071 China; 7 Department of Engineering and Technology, Institute of Oceanology, Chinese Academy of Sciences, Qingdao, 266071 China

**Keywords:** Marine biology, Community ecology, Carbon cycle

## Abstract

Zooplankton can affect and regulate the biological carbon pump in the biogeochemical cycles of marine ecosystems through diel vertical migration (DVM) behaviour. The diel vertical distribution and migration of a zooplankton community were studied at a continuous survey station in the Caroline Seamount area of the western tropical Pacific Ocean. Using a MultiNet sampling system, 346 zooplankton species/taxa were collected and identified. The vertical distribution patterns of abundance and composition of the zooplankton community differed between daytime and nighttime. The highest biodiversity index occurred in the 100–200-m ocean depth layer, but some zooplankton species remained in the deep-water layer below 300 m. The DVM patterns of the various dominant species differed, even when the species belonged to the same order or family. Dissolved oxygen and seawater temperature were the main environmental factors affecting the diel vertical distribution of the zooplankton community. The oxygen minimum zone was identified as performing the dual role of “ecological barrier” and “refuge” for zooplankton. The active carbon flux mediated by the zooplankton DVM in the Caroline Seamount area was 14.5 mg C/(m^2^·d). Our findings suggest that zooplankton DVM can affect and mediate the biological carbon pump in the Caroline Seamount area.

## Introduction

Zooplankton dominate marine organism biomass and are ubiquitous throughout the world's oceans. As the largest portion of marine organism biomass, they are an important indicator of global climate change and ocean circulation, and play a key role in the marine food chain^1,2^. Zooplankton feed on phytoplankton and serve as an important food source for higher trophic levels in pelagic ecosystems. They are also crucial for the flow of energy and materials as well as for the global biogeochemical cycle of oceans^3–5^. Most zooplankton inhabit deeper water during the day and ascend to the upper layers at night, a pattern termed diel vertical migration (DVM)^6^. The DVM of zooplankton is regarded as a vehicle for carbon export in the marine carbon cycle^7,8^. However, due to the different adaptive characteristics of zooplankton, not all zooplankton show obvious DVM behaviours. Ostracods always showed pronounced DVM in the open sea^9^. Only few copepod species (*Eucalanus elongatus*, *Scolecithricella dentata*, *Corycaeus furcifer*, and *Pleuromamma gracilis*) showed significant DVM behaviours in the Mediterranean Sea^10^. The most abundant euphausiid species, *Euphausia hanseni*, showed pronounced DVM behaviour in the highly productive northern Benguela upwelling system^11^. This difference in DVM behaviours could facilitate the niche overlap in zooplankton community.

The western tropical Pacific Ocean (WTPO) is a key region for oceanographic research. It has unique ecosystems due to its extraordinary geography and highly complex current system^12–14^. Seamounts are generally defined as isolated and uplifted terrain below the seawater surface with summit depths of at least 1000 m above the seafloor^15–17^. Globally, there are more than 170,000 seamounts, but only a few have been well studied^16,17^. Because of their specific geographical characteristics and hydrological conditions, seamounts provide unique habitats and environmental conditions for marine organisms, potentially supporting high biodiversity and unique biological communities^18,19^. The Global Census of Marine Life on Seamounts (CenSeam), established in 2005, studies seamount ecosystems to determine their role in the biogeography, biodiversity, productivity, and evolution of marine organisms^20^. Although numerous studies have been conducted on benthic organisms and biogenic elements in seamount areas^16,21,22^, few have focused on zooplankton community structure, let alone the DVM of zooplankton communities in seamount areas. The DVM behaviour and vertical spatial structure of sound-scattering layers at two seamounts in the Azores and in surrounding open-waters were examined^23^. Spatial distribution patterns of microzooplankton at two shallow seamounts in the subtropical and tropical NE Atlantic were reported^24^. The vertical distribution (0–3000 m) of the zooplankton community in the western tropical North Pacific was studied based on ZooScan measurements with samples obtained using a MultiNet system^25^. Different vertical distribution of zooplankton community between North Pacific Subtropical Gyre and Western Pacific Warm Pool was reported^26^.

In this study, we report the population abundance, community structure, DVM pattern, carbon biomass, and carbon flux of the zooplankton community in the Caroline Seamount area. We systematically describe the effects of the environmental factors, especially dissolved oxygen concentration, on the zooplankton community in the seamount area to further understand the carbon flux mediated by zooplankton in the Caroline Seamount area of the WTPO.

## Results

### Environmental conditions

**Figure 1:**
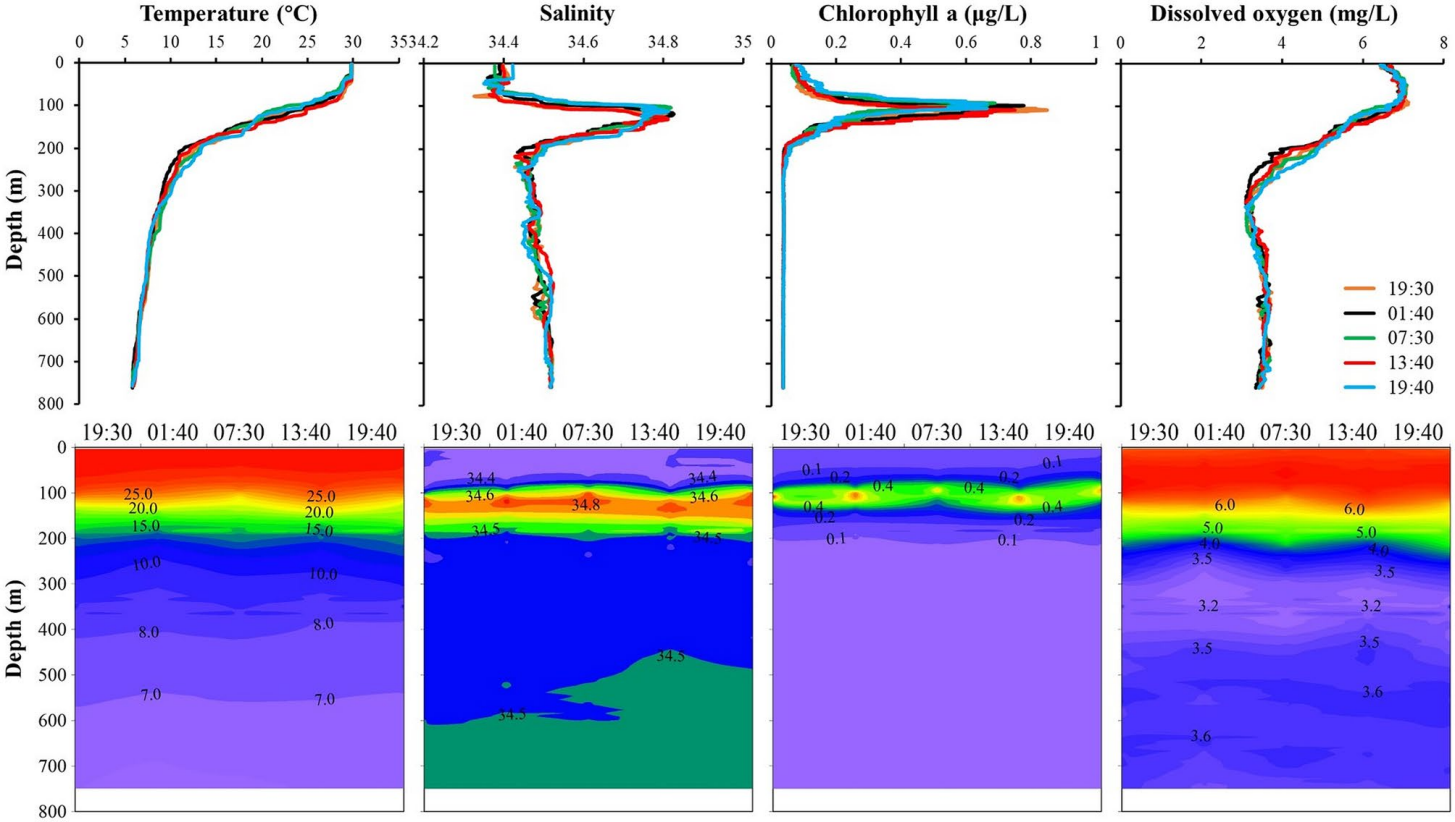
The vertical distribution of the environmental factors in the Caroline Seamount area on June 2 to 3, 2019.

The diel vertical variations in environmental factors (ST: seawater temperature, SS: seawater salinity, CHL: chlorophyll fluorescence, DO: dissolved oxygen) at survey station B3 are shown in Fig. 1. There were no statistically significant differences among the five samples for any of the environmental factors examined (one-way ANOVA, *P* < 0.01). The water column was stratified at all sampling times. The ST decreased gradually with increasing depth. The thickness of the seawater surface to mixed layer exceeded 100 m, with ST > 25.0 °C, SS < 34.5, CHL > 0.25 μg/L, and DO > 6.50 mg/L. The highest and lowest STs were 29.88 °C and 5.79 °C, respectively. A thermocline occurred at a depth of 120–200 m. The ST decreased rapidly from > 22.0 to < 12.0 °C in the thermocline. From the surface to a depth of 200 m, both SS and CHL generally increased and then decreased with depth. A halocline occurred in the 75–200-m layer. The maximum and minimum SS values were 34.83 and 34.33 PSU, respectively. The maximum CHL was 0.85 μg/L. The deep chlorophyll maximum (DCM) layer was detected at 100–120 m. The DO increased slightly from the sea surface to a depth of 100 m and decreased rapidly from 100 m to the deeper layer. The oxygen minimum zone (OMZ) was found in the 300–400-m layer, and the lowest DO was less than 3.0 mg/L (Fig. 1).

### Species composition and diversity of the zooplankton community

From June 2 to 3 2019, we collected and identified 346 zooplankton species/taxa in the Caroline Seamount area, including 163 species of Copepoda, 36 species of Cnidaria, 32 species of pelagic ostracods, 26 taxa of pelagic larvae, 21 species of Amphipoda, 16 species of Chaetognatha, 12 species of Euphausiacea, 12 species of tunicates, 12 species of planktonic pteropods, 4 species of Polychaeta, 3 species of Decapoda, 3 species of Heteropoda, 2 species of Mysidacea, 2 species of Ctenophora, 1 species of Isopoda, and 1 species of Cladocera. The Sørensen’s coefficient of similarity (*SCS*) values between the five sampling times were high, ranging from 71.1 to 80.8%, and the average *SCS* of all zooplankton communities sampled was 76.00%. Therefore, the five zooplankton samples collected at different times can be regarded as belonging to the same zooplankton community of the Caroline Seamount area.

**Figure 2:**
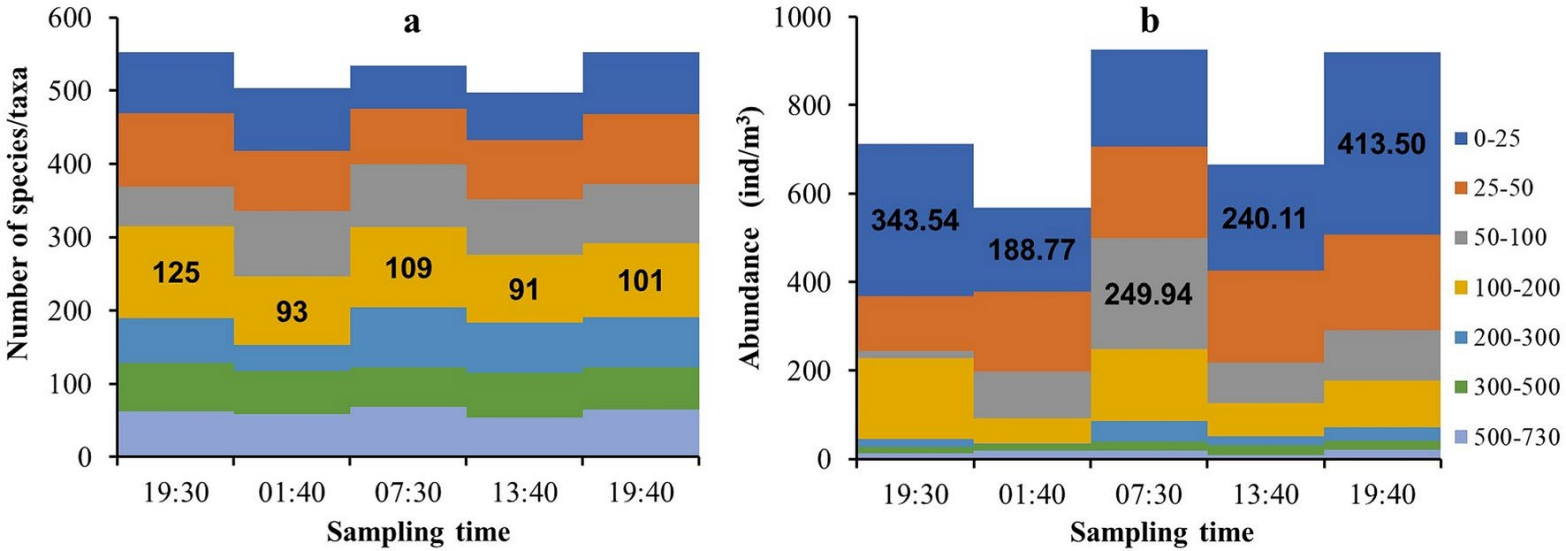
The diel vertical variations of zooplankton species/taxa (**a**) and abundances (**b**) in the Caroline Seamount area on June 2 to 3, 2019. The data label in bold black font indicates the highest value.

The numbers of species/taxa collected throughout the water column at the five sampling times (19:30, 01:40, 07:30, 13:40, and 19:40) were 247, 211, 215, 201, and 217, respectively. There were 127 common species/taxa among the five sample collections. At each sampling time, the 100–200-m layer had the highest numbers of zooplankton species/taxa. The diel vertical variation in zooplankton species/taxa was not obvious, but the number of zooplankton species/taxa collected from the whole water column was lowest at 13:40 and highest at 19:30 (Fig. 2a). The total zooplankton abundances for the whole water column at the five sampling times (19:30, 01:40, 07:30, 13:40, and 19:40) were 54.72, 40.88, 71.49, 48.79, and 71.33 ind/m^3^, respectively. However, the distribution of the highest species/taxa number and abundance in different water layers showed different trends. For all sampling times except 07:30, the highest abundance was found in the 0–25-m layer (Fig. 2b). The highest values of zooplankton community diversity did not coincide with those of zooplankton abundances.

**Figure 3:**
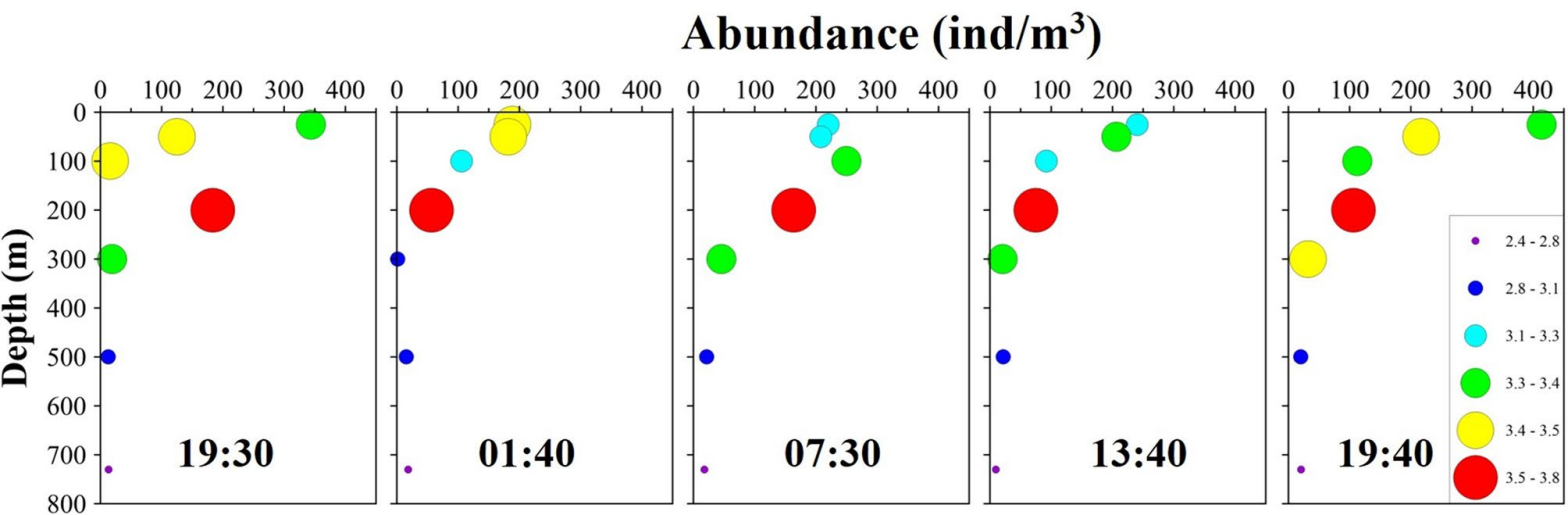
The diel vertical variations of zooplankton abundance and diversity (*H'*) in the Caroline Seamount area on June 2 to 3, 2019.

The Shannon–Wiener index (*H'*) of the zooplankton community for the whole water column at the five sampling times were 3.93 (19:30), 3.78 (01:40), 3.79 (07:30), 3.77 (13:40) and 3.83 (19:40). The values of *H'* increased and then decreased from the surface to the bottom layer. The highest *H'* values of the five sampling times were all ≥ 3.50 and occurred in the 100–200-m layer, which was consistent with the location of the thermocline and DCM (Fig. 3). However, the water layer with the highest zooplankton biodiversity was not consistent with the water layer with the highest abundance. Seven dominant species were identified at the survey station. According to the dominance index from high to low, these species were *Clausocalanus furcatus* (*Y* = 0.110), *Oithona plumifera* (*Y* = 0.100), *Clausocalanus arcuicornis* (*Y* = 0.057), *Farranula gibbula* (*Y* = 0.044), *Oncaea venusta* (*Y* = 0.032), *Acartia negligens* (*Y* = 0.029), and *Oncaea media* (*Y* = 0.027). These dominant species were all small copepods with a body length of less than 2 mm.

### Vertical distribution of the zooplankton community structure

Among the five sampling times, the proportions of zooplankton above 200 m and 300 m to the total number of zooplankton communities were 78.0% and 84.0%, respectively. A total of 80.0% of zooplankton individuals were distributed in the water layer above 200 m during the nighttime. This proportion decreased to 75.4% during the daytime. However, the proportions in the 0–300-m water layer during the nighttime and daytime were similar (84.0% and 83.9%, respectively). The proportion above 200 m at the 01:40 sampling time was higher than that at the 13:40 sampling time.

According to the location of the OMZ, the seven sampled water layers are grouped into three layers: the upper layer (0–300-m), the OMZ located layer (300–500-m), and the bottom layer (500–730-m). Among the 127 common species/taxa found at the five sampling times, 111 zooplankton species/taxa were mainly distributed in the upper layer, with the highest percentage of the total. More than 80% of the individuals of 89 zooplankton species/taxa were found in the upper layer during the survey period. Furthermore, 34 zooplankton species/taxa were always found in the upper layers and never crossed the OMZ layer during the five sampling times. However, 6 zooplankton species (*Subeucalanus mucronatus*, *Chirundina streetsii*, *Nematoscelis atlantica*, *Lensia multicristatoides*, *Spinocalanus spinosus*, and *Rhincalanus cornutus*) were mainly distributed in the OMZ located layer. More than half of the individuals of the first three species (*S. mucronatus*, *Chirundina streetsii*, and *N. atlantica*) were distributed in the OMZ located layer. In addition, 30%-50% of the individuals of another 11 species/taxa were distributed in the OMZ located layer (Table 1).

**Table 1:** The quantity percentage in different sampling layers of zooplankton species/taxa mainly distributed in the OMZ located layer and bottom layer in the Caroline Seamount area on June 2 to 3, 2019.

Species/taxa	0–300-m	300–500-m	500–730-m	Main distribution layer
*Subeucalanus mucronatus*	34.9%	**62.0%**	3.1%	OMZ located layer
*Chirundina streetsii*	10.0%	**52.5%**	37.5%	
*Nematoscelis atlantica*	41.4%	**51.7%**	6.9%	
*Lensia multicristatoides*	8.3%	**45.9%**	45.8%	
*Spinocalanus spinosus*	33.4%	**43.1%**	23.5%	
*Rhincalanus cornutus*	31.9%	**38.9%**	29.2%	
*Lucicutia magna*	1.4%	1.4%	**97.2%**	Bottom layer
*Metridia brevicauda*	0%	8.5%	**91.5%**	
*Undeuchaeta plumosa*	15.6%	13.0%	**71.4%**	
*Eucalanus elongatus*	0%	33.3%	**66.7%**	
*Mormonilla minor*	12.8%	23.6%	**63.6%**	
*Temoropia mayumbaensis*	26.9%	11.6%	**61.5%**	
*Oncaea gracilis*	2.1%	36.8%	**61.1%**	
*Scaphocalanus echinatus*	37.3%	12.0%	**50.7%**	
*Aegisthus mucronatus*	28.1%	29.8%	**42.1%**	
*Scolecithricella sp*	36.0%	24.6%	**39.4%**	

The bold font indicates the maximum value of the quantity percentage in different sampling layers of zooplankton species/taxa.

### DVM patterns of the zooplankton species

**Figure 4:**
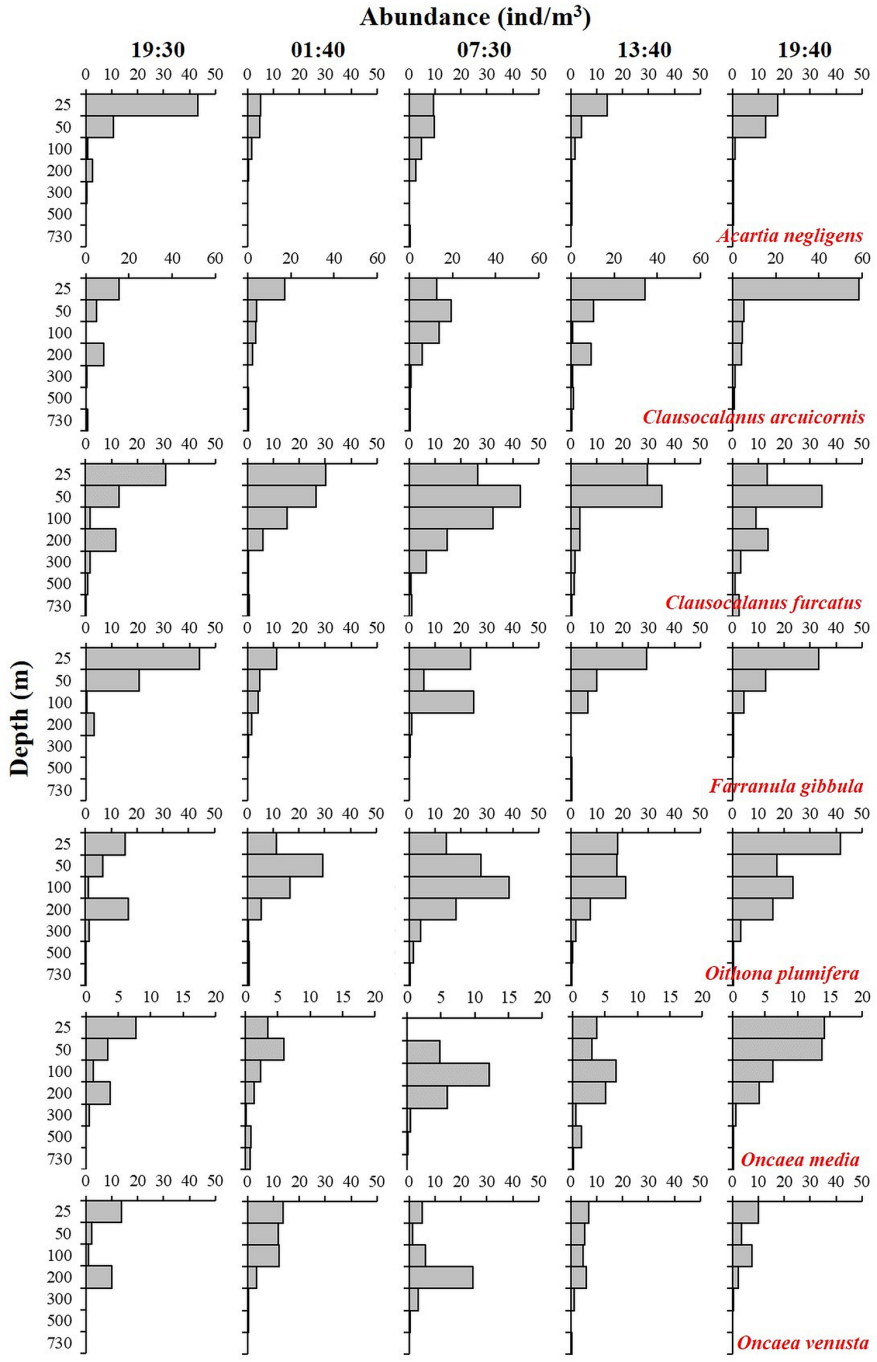
Diel vertical distribution in numerical abundance (ind/m^3^) of the dominant zooplankton species in the Caroline Seamount area on June 2 to 3, 2019.

**Figure 5:**
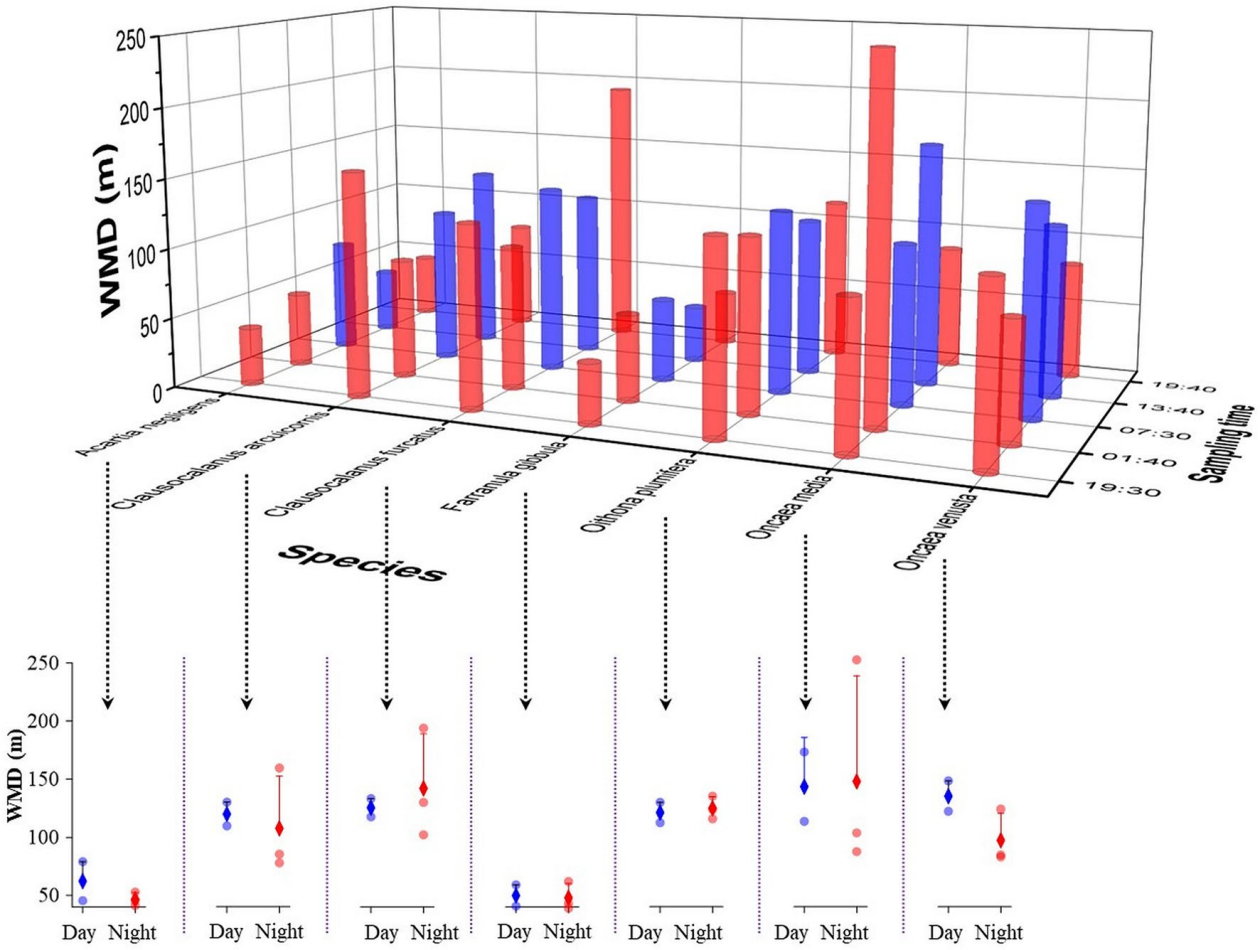
The WMDs of the dominant zooplankton species and the differences between daytime (Day) and nighttime (Night) in the Caroline Seamount area on June 2 to 3, 2019.

The vertical distribution of abundances and WMDs of the dominant species are shown in Figs. 4 and 5. The water layer with the highest abundances of *A. negligens* and *F. gibbula* was the same. The average WMDs of both species were the shallowest among the seven dominant species. There was little difference in the WMD between daytime and nighttime for the two species. Thus, neither *A. negligens* nor *F. gibbula* showed obvious DVM behaviour. The average WMD of *C. furcatus* was deeper than that of *C. arcuicornis*. The diel differences in WMD between the two *Clausocalanus* species were similar, but their highest abundance in the water layers was not consistent. The water layer with the highest abundance of *C. arcuicornis* was the same as that of *A. negligens* and *F. gibbula*, with all three species descended from the 0–25-m layer to the 25–50-m layer by the 07:30 sampling time. Therefore, the two *Clausocalanus* species did not show an obvious DVM pattern. Although the layer with the highest abundance of *Oithona plumifera* and *O. media* was the 50–100 m layer during the daytime and the shallower layer above 50 m during the nighttime, the differences in WMDs between daytime and nighttime for the two species were not significant (3.8 m and 4.5 m). Among the seven dominant species, only *O. venusta* showed an obvious DVM pattern, staying in the deeper water layer during the daytime and migrating to the upper layers at the nighttime. First, the difference in the value of WMDs between daytime and nighttime for *O. venusta* was the largest (38.0 m). Second, the high abundances of *O. venusta* were mainly distributed in the 0–25-m layer at the 19:30, 01:40, and 19:40 sampling times and in the 100–200-m layer at the 07:30 and 13:40 sampling times.

Additionally, 33 zooplankton species/taxa could migrate vertically and cross the OMZ layer. They included Copepoda (22 species), pelagic larvae (3 species/taxa), Cnidaria (2 species), Amphipoda (1 species), Chaetognatha (1 species), Decapoda (1 species), Euphausiacea (1 species), ostracods (1 species), and fish larvae. Among them, three copepods (*Chirundina streetsii*, *Euchirella curticauda*, and *Oncaea* spp.) and one euphausiid (*Nematoscelis atlantica*) showed an obvious DVM pattern, in which the difference in the WMD values between daytime and nighttime were 265.4, 234.2, 61.3, and 207.6 m, respectively.

### The vertical distribution of carbon biomass and the active carbon flux in the zooplankton community

**Figure 6:**
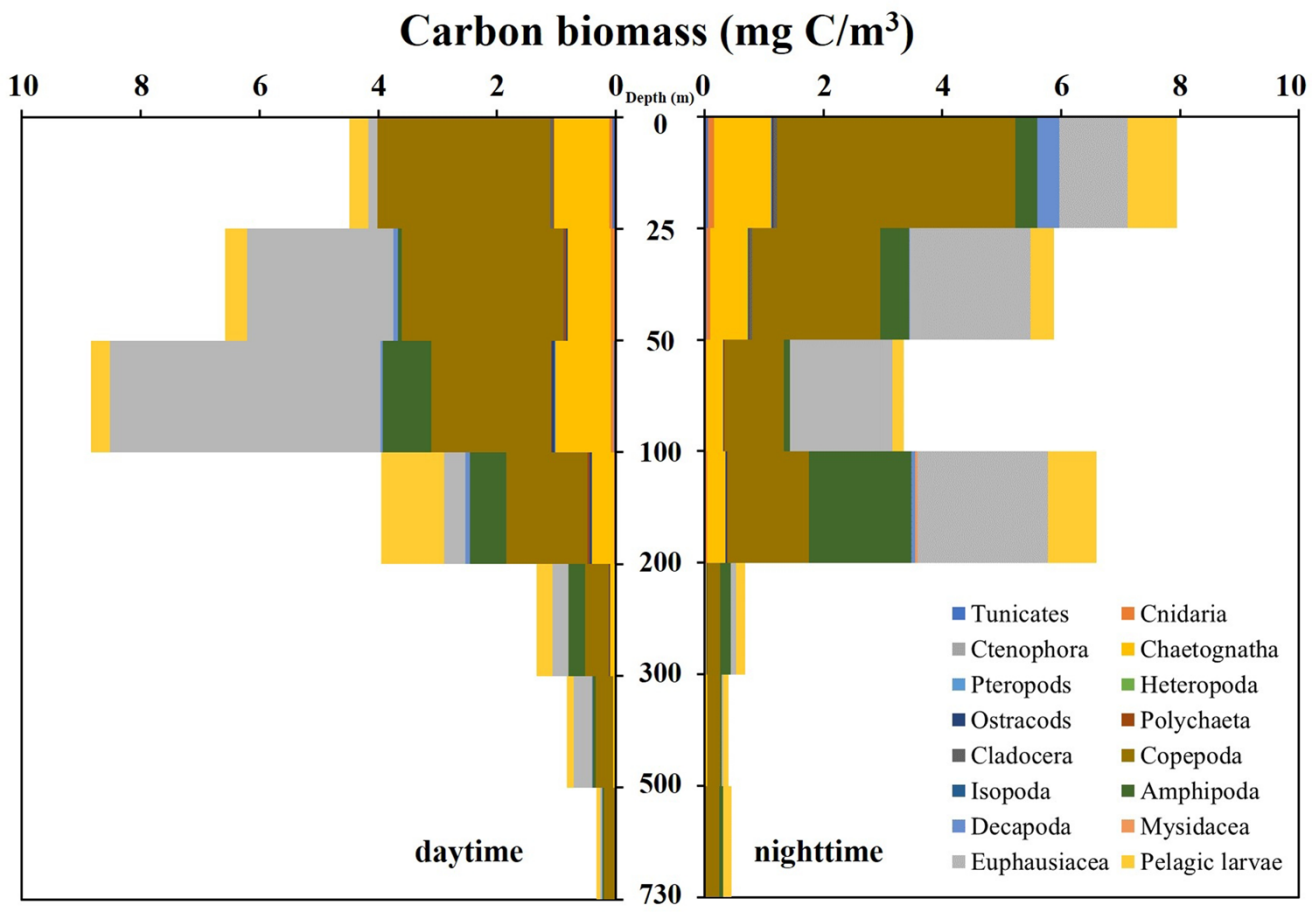
The diel vertical variations of zooplankton carbon biomass in the Caroline Seamount area on June 2 to 3, 2019.

The total CB of the zooplankton community in the water column at the five sampling times was 24.35 (19:30), 20.22 (01:40), 34.31 (07:30), 18.38 (13:40) and 31.38 (19:40) mg C/m^3^. The total CB in the water column at the 01:40 and 13:40 sampling times was significantly lower than that at other sampling times, which is consistent with the vertical distribution trend of zooplankton species/taxa number and abundance. The vertical distribution and composition of the zooplankton community CB during the daytime and nighttime are shown in Fig. 6. The highest CB in the water column was found in the top layer during the nighttime, and it was distributed in the 50–100-m layer during the daytime. From nighttime to daytime, the high zooplankton CB values migrated from the 50–100-m layer to the 0–25-m and 100–200-m layers (Fig. 6). The total zooplankton CB of the whole water column during the daytime and nighttime were similar, at 25.32 and 26.34 mg C/m^3^, respectively. The total zooplankton CB in the water layer above 200 m made up 93.9% of the total water column CB during the nighttime. However, this proportion was only 90.5% during the daytime. During the nighttime and daytime, 1.5–1.9% (average value: 1.6%) and 2.3–4.7% (average value: 3.2%) of the zooplankton CB was found in the OMZ located layer, respectively. The proportion of zooplankton CB below the 300 m layer during the daytime (4.4%) was higher than that during the nighttime (3.4%). Thus, approximately 0.86 mg C/m^3^ zooplankton CB was transported from the 0–200-m layer to deep layers over the diurnal period. Furthermore, the OMZ located layer contained 0.36–0.87 mg C/m^3^ zooplankton CB across the five sampling times.

Based on the vertical distribution of the zooplankton community, we chose 200 m as the threshold depth for the daily biomass of zooplankton migrators. The diel-migrating zooplankton biomass in the 0–200-m layers was 76.3 mg C/m^2^. The migrating zooplankton CF was 14.5 mg C/(m^2^ d) at station B3 in the Caroline Seamount area.

### Relationship between the zooplankton community and environmental factors

**Figure 7:**
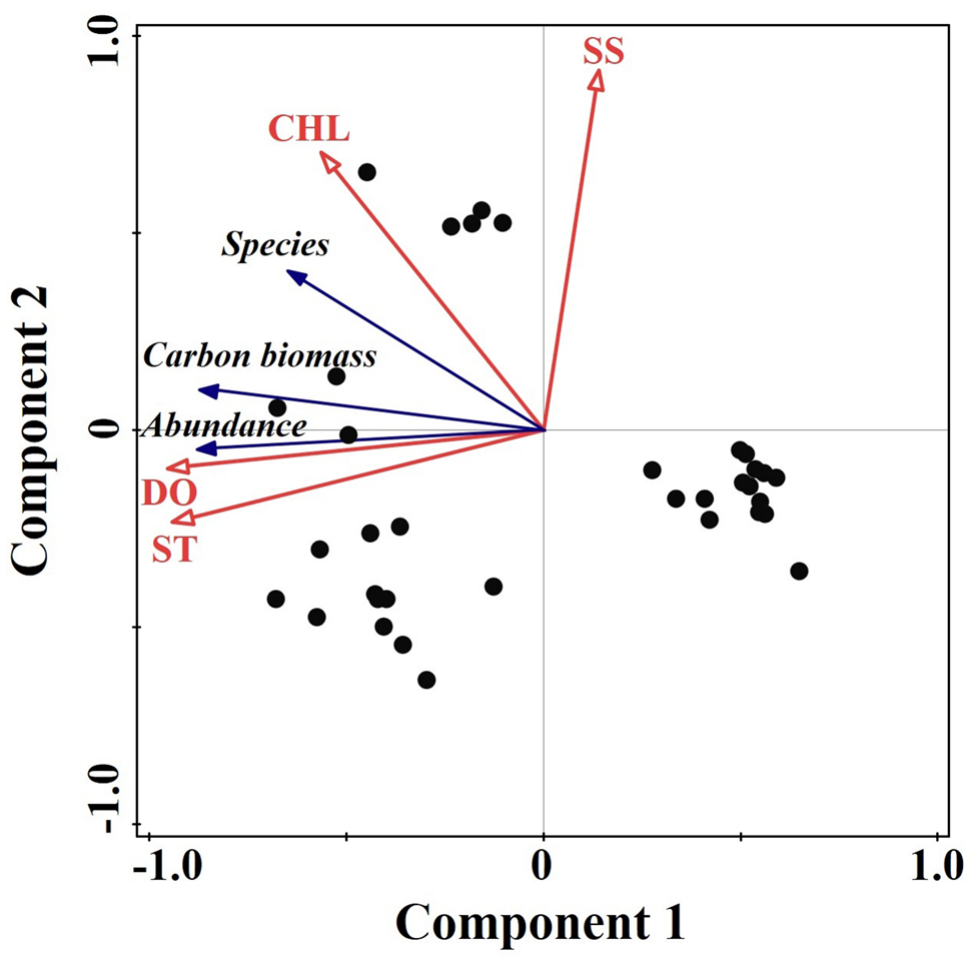
PCA scatter diagrams of zooplankton community parameters and environmental factors (ST, seawater temperature; SS, seawater salinity; CHL, chlorophyll fluorescence; DO, dissolved oxygen) of different sampling layers in the Caroline Seamount area on June 2 to 3, 2019.

Pearson correlation analyses showed that both the number of zooplankton species/taxa and CB were positively correlated with ST, CHL, DO, and SS (*P* < 0.05). Similarly, the Shannon–Wiener index was significantly positively correlated with ST, CHL, and DO (*P* < 0.01). The zooplankton species/taxa number, abundance, and carbon biomass, and the environmental parameters of the different sampling layers and times were analysed by PCA (Fig. 7). The cumulative percentage of variance for the initial eigenvalues of the two principal components was 85.04% of the total variance explained by the seven components (species, abundance, CB, ST, SS, CHL, and DO). The total variance of principal component 1 was 60.16%. In the principal component 1 matrix, the PCA loading value (absolute value) of DO was the highest (− 0.94), and that of SS was the lowest (0.14). The PCA loading values of other components were − 0.91 (ST), − 0.90 (CB), − 0.86 (abundance), − 0.71 (species), and − 0.64 (CHL). ST and DO were the two environmental variables with the greatest influence during the survey period. The influence of ST and DO was similar, showing a significant positive correlation with the abundance and carbon biomass of the zooplankton community. Similarly, the zooplankton species/taxa number was positively affected by CHL. However, the correlation between the three characteristic parameters of the zooplankton community and SS was not obvious.

BIO-ENV analysis is based on determining the Spearman’s rank correlation coefficient (*ρ*) between the biological and environmental similarity matrices. A *ρ* value ranging from 0 to 1 represents no match to a perfect match between the two matrices. The indicators of the changes in the zooplankton community at the five sampling times differed (Table 2). These indicators were analysed with the BIO-ENV procedure, which identified ST as the best indicator of zooplankton community changes at sampling times 19:30 (*ρ* = 0.71), 13:40 (*ρ* = 0.96), and 19:40 (*ρ* = 0.95). A combination of ST and DO was the best two-variable indicator at sampling times 01:40 (*ρ* = 0.84) and 07:30 (*ρ* = 0.94). At all sampling times, ST had the highest *ρ* value, but DO became the most important indicator at nighttime. Based on the results of the PCA and BIO-ENV analyses, ST and DO were the primary environmental factors affecting the vertical distribution of the zooplankton community.

**Table 2:** Results of BIO-ENV analysis between environmental factors (ST, SS, CHL, and DO) and zooplankton community in the Caroline Seamount area on June 2 to 3, 2019.

19:30		01:40		07:30		13:40		19:40	
*ρ*	Factors	*ρ*	Factors	*ρ*	Factors	*ρ*	Factors	*ρ*	Factors
0.71	ST	0.84	ST, DO	0.94	ST, DO	0.96	ST	0.95	ST
0.69	ST, DO	0.80	ST	0.92	ST	0.95	ST, DO	0.93	ST, DO
0.66	DO	0.78	DO	0.90	DO	0.82	DO	0.88	DO
0.40	ST, CHL	0.56	CHL, DO	0.67	ST, CH	0.56	ST, CH	0.70	ST, CH
0.38	CH, DO	0.51	ST, CHL	0.59	CHL, DO	0.54	ST, SS	0.68	CHL, DO
0.30	ST, SS	0.35	ST, SS	0.56	SS, DO	0.51	CHL, DO	0.48	ST, SS

*ρ* indicates the Spearman correlation coefficient between zooplankton community and environmental factors.

## Discussion

Zooplankton DVM is a common and vital ocean phenomenon that not only changes the structure and conditions of the zooplankton community, but also affects the distribution and behaviour of higher trophic level organisms such as the fish that feed on zooplankton^27^. Many studies have found that zooplankton species could exhibit DVM behaviours in seamount areas^28–31^. There are four zooplankton DVM patterns: nocturnal (the most common form), twilight, reverse, and tidal^32^. As indicated by the differences between nighttime and daytime WMD values, the DVM patterns of the seven dominant species in the Caroline Seamount area were different. The different DVM patterns of the dominant species explained the differences between the highest abundance centre of the zooplankton community at the 07:30 sampling time and the other four sampling times. *Oncaea venusta* showed the most obvious nocturnal DVM behaviours. The mean WMD of *Clausocalanus furcatus* at nighttime was lower than that in the daytime. However, according to the abundances of each layer and the WMDs of the five sampling times, *Clausocalanus furcatus* showed the tidal pattern DVM, which reflects the semidiurnal tide cycle. Additionally, based on the DVM distance of zooplankton between daytime and nighttime, there was no obvious DVM behaviours in the other dominant species (*A. negligens*, *C. arcuicornis*, *F. gibbula*, *O. media*, and *Oithona plumifera*). Although *Chirundina streetsii* and *Nematoscelis atlantica* were mainly distributed in the OMZ located layer, both species, as well as *Euchirella curticauda* and *Oncaea* spp., showed an obvious nocturnal DVM pattern. Comparing the results for the highest abundance water layer with those for WMD, we suggest that the WMD approach is more accurate and suitable than the highest abundance water layer for studying zooplankton DVM patterns. The DVM of zooplankton is affected by many factors, including light^33,34^, the presence of a thermocline^35^ or halocline^36^, dissolved oxygen^37,38^, food sources^39^, predation^40^, and currents^41^. In our research, Pearson correlation analysis results showed that the zooplankton community diversity was positively correlated with four environmental factors (ST, SS, CHL, and DO). The highest numbers of zooplankton species/taxa were distributed in the water layer with high ST, SS, DO, and CHL. However, zooplankton abundance was positively correlated only with ST and DO. The abundances of the seven dominant species were all positively correlated with the DO concentrations. The BIO-ENV analysis also showed that ST and DO were the best indicators of the zooplankton community in the Caroline Seamount area at the five sampling times.

With the increasing global ocean deoxidation and the global expanding of the OMZs^42^, the OMZs were found in the western Pacific Ocean^25^ and seamount areas of the WTPO^43–45^, including the Caroline Seamount. The presence of an OMZ is an important physiological limitation for zooplankton with DVM behaviour. Hypoxia can affect the respiratory metabolism of zooplankton and relatively few food sources are available in the OMZ. Therefore, the OMZ may be a “barrier” that blocks zooplankton migration to deeper layers to avoid predation^46^. However, many studies have also found that some zooplankton species occur predominantly in the OMZ. The findings in these studies point to the OMZ as a “refuge” with low oxygen concentrations that keeps predators away^37,47–49^. Near seamount Volcano 7 area in the eastern tropical Pacific, the maximum values for zooplankton biomass and copepod abundance occurred in the thermocline zone, and the vertical distribution of copepod abundance was related to DO. Some zooplankton species, such as *Clausocalanus* spp., *Oncaea* spp., and *Oithona* spp., were found to cross the OMZ by vertical migration^37^. The DVM characteristics of euphausiids, which have relatively strong mobility, were demonstrated as they moved into the OMZ of the Humboldt Current Ecosystem^38^. The findings of the study showed that the OMZ determined the migration time, migration intensity, and residence time in the OMZ of different euphausiid species. *Euphausia hanseni* could regularly cross the thermocline and retreat again to the OMZ^11^. Decapods are able to regulate their respiration to live aerobically in the OMZs across the tropical and subtropical Atlantic Ocean^50^. In our study, zooplankton abundances above the OMZ layer accounted for more than 94.0% of the total abundance. Zooplankton abundance was the lowest in the OMZ located layer at the 19:30 and 19:40 sampling times, and in the bottom layer at the 07:30 and 13:40 sampling times. The lowest zooplankton abundance occurred in the 200–300-m layer at the 01:40 sampling time. Therefore, for most zooplankton in the Caroline Seamount area, the OMZ is an ecological barrier that prevents DVM through the entire water column. For example, individuals of all dominant species did not cross the OMZ and were always distributed in the layers above 300 m depth. However, 33 species in eight taxa could cross the OMZ layer. Some euphausiids could pass through the OMZ to reach the deepest layer, but they mainly stayed in the layers above 300 m, especially the 100–200-m layer where the DCM was located. At the 07:30 and 13:40 sampling times, six euphausiid species were collected in the 300–500-m layer where the OMZ was located. Moreover, we also found that four copepod species, one euphausiid species and one cnidaria species were mainly distributed in the OMZ located layer. We suggest that these species have adapted to the hypoxic environment and regard the OMZ as a refuge. This pattern indicates that some zooplankton live in the OMZ to avoid predation or due to ecological niche competition.

Due to the influence of various thermoclines and topography, the nutrients in the upper and deep layers of the tropical Pacific Ocean are normally not exchanged rapidly. The DVM of zooplankton could improve the vertical carbon flux and contribute to the function of the biological carbon pump in the ocean^51^. The CF of zooplankton varies in different regions and is generally higher in eutrophic and mesotrophic regions than in oligotrophic regions^50^. Our study area, the Caroline Seamount, is located in the WTPO, a typical “oligotrophic” region. In the Western Pacific Ocean, the zooplankton CF in oligotrophic regions ranges from 1.8 to 5.3 mg C/(m^2^·d)^26,52–54^. In the mesotrophic subarctic Pacific, the active carbon flux (CO_2_ and DOC) transport by migrator respiration and excretion was found to be 16–46 mg C/(m^2^·d) measured by sediment traps at 150 m, compared with 2–8 mg C/(m^2^·d) in the oligotrophic North Pacific subtropical gyre^55^. Using a MOCNESS multiple net system (mesh size: 333 μm, mouth opening: 1 m^2^) and the electron transfer system activity method, zooplankton metabolic rates were studied at two seamounts (Seine: summit depth similar to 170 m; Sedlo: summit depth similar to 750 m) in the northeast Atlantic. The depth-integrated (0–150 m) median respiratory carbon demand of the zooplankton community estimated from day and night hauls was 2.1 mg C/(m^2^·d) at the Seine Seamount and 2.9 mg C/(m^2^·d) at the Sedlo Seamount^56^. The taxonomic composition and the respiratory carbon demand of the zooplankton community were estimated using a 1-m^2^ MOCNESS (333 μm mesh size) and a 0.25-m^2^ MultiNet (200 μm mesh size) at Ampere and Senghor, two shallow seamounts in the subtropical and tropical northeast Atlantic. The mean respiratory carbon demand in the epipelagic zone (upper 1000 m) was 61.4 mg C/(m^2^·d) for Senghor and 9.6 mg C/(m^2^·d) for Ampere^57^. Seamounts can block underwater currents to form upwellings and Taylor columns. They can bring sufficient nutrients to the upper sea area, which may enhance primary productivity, thereby increasing biodiversity and biomass in the seamount area. This phenomenon was called the “seamount effect”^23,58^. An upwelling was found in the euphotic zone of Caroline M5 seamount area, causing accumulations of nutrients and phytoplankton around the seamount and forming a “seamount effect”^59^. Although Senghor seamount as a hotspot for meroplanktonic larvae, suggesting a retention potential that results in significantly enhanced larval abundance in the seamount waters as compared with the open ocean, no clear evidence of “seamount effects” resulting in enhanced microzooplankton biomass were detected^60^. Our results show that the zooplankton community transported 0.40 mg C/m^3^ CB from the upper layer to the OMZ layer through the DVM behaviours of the 33 zooplankton species/taxa. The zooplankton CB above 200 m in the Caroline seamount area during the daytime was lower than that during the nighttime, which indicates that a lot of zooplankton carbon was transported to the water layer below 200 m and deeper layers by the DVM of the zooplankton. The CF mediated by zooplankton DVM in the Caroline Seamount area was 14.5 mg C/(m^2^·d) at 0–200 m, which is similar to that of other seamount areas in other oligotrophic regions, but higher than that in the Western Pacific Ocean. Our other study shows that both abundance and biomass of zooplankton community in the Caroline Seamount area were higher than those in the Yap and Kocebu Seamount areas of the western Pacific Ocean (unpublished data). So, the zooplankton CF in the Caroline Seamount area was relatively high. The geological conditions of the Caroline Seamount and the Sedlo Seamount are similar, but the zooplankton CF of the former is much higher than that of the latter. Therefore, we suggest that the DVM behaviour of zooplankton plays an important role in the biological carbon pump of the Caroline Seamount area.

## Methods

### Survey times, study area, and zooplankton sampling

**Figure 8:**
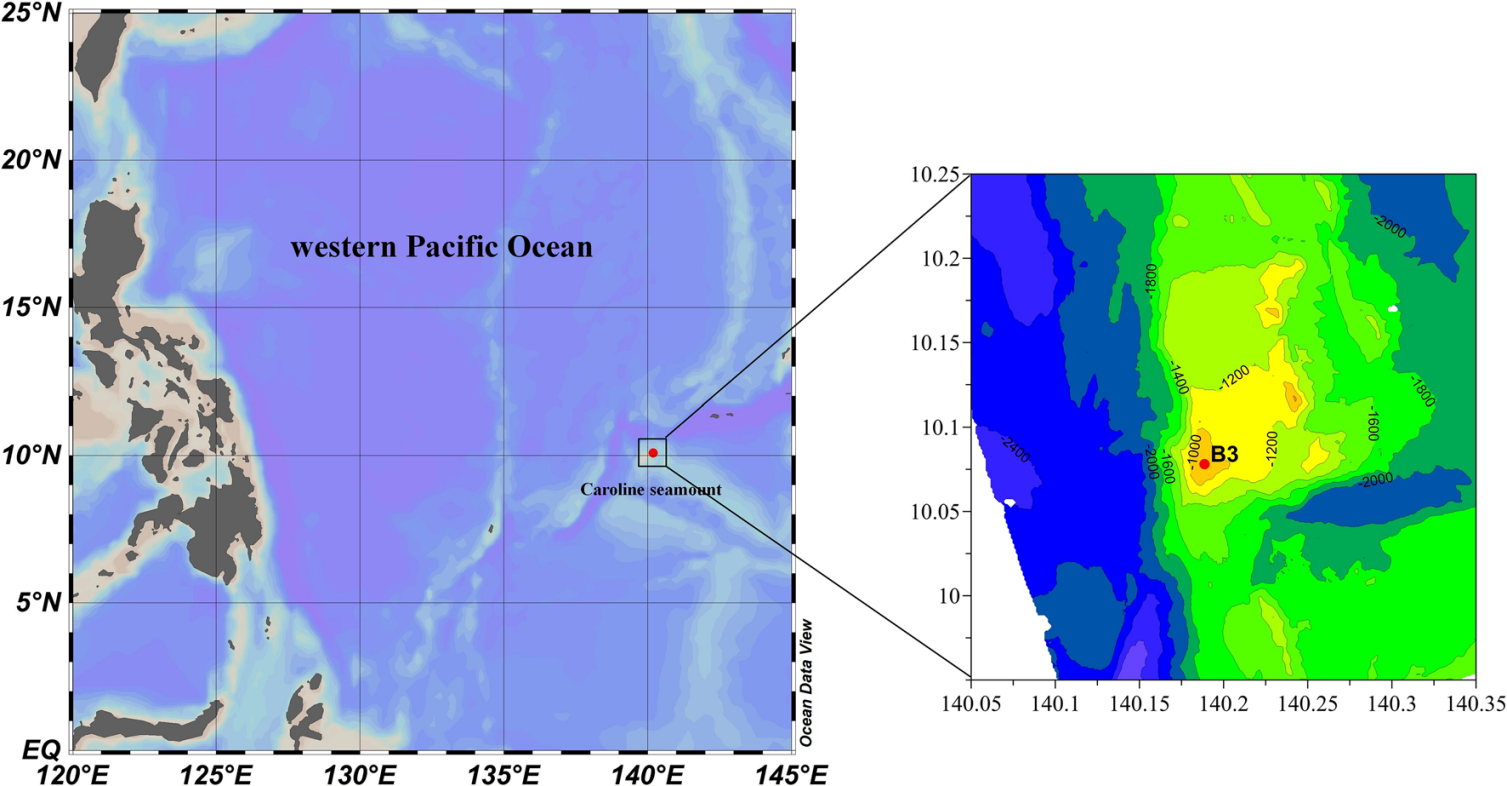
Sampling station B3 in the Caroline Seamount of the western tropical Pacific Ocean on June 2 to 3, 2019.

The survey was conducted in the Caroline Seamount area of the WTPO onboard the R/V “*KEXUE*” from May 26 to June 12, 2019. A map of the surveyed area was created using bathymetric data collected using an R/V-mounted multibeam echo sounder system. Survey station B3 is located in the southwestern part of the Caroline Seamount area (140°11′19″E, 10°4′41″N; depth: 810 m; Fig. 8). The station map and topographic map of the seamount area are drawn by Ocean Data View 4 (http://odv.awi.de/) and Golden Software Surfer 14 (www.goldensoftware.com). Zooplankton were collected with a Max MultiNet (mesh size: 200 μm, mouth opening: 0.50 m^2^, HYDRO-BIOS, Germany). Sampling was conducted from near the bottom to the surface at a rate of approximately 0.8–1.0 m/s every 6 h on June 2 to 3, 2019. Five sampling operations were carried out at 19:30 on June 2, 01:40 on June 3, 07:30 on June 3, 13:40 on June 3, and 19:40 on June 3. We considered 19:30, 01:40, and 19:40 to be nighttime samplings, and 07:30 and 13:40 to be daytime samplings. During each sampling event, zooplankton were collected from seven water layers: 0–25 m, 25–50 m, 50–100 m, 100–200 m, 200–300 m, 300–500 m, and 500–730 m. The 35 zooplankton samples were preserved in 5% buffered formalin seawater solution immediately upon collection. Environmental parameters, including seawater temperature (ST), salinity (SS), chlorophyll fluorescence (CHL), and dissolved oxygen (DO) of the sampling station, were measured simultaneously during zooplankton collection using a CTD integrated with MultiNet sampling system.

### Zooplankton abundance, carbon biomass and active carbon flux

In the laboratory, zooplankton samples were identified and counted with a stereoscopic microscope (Nikon SMZ745, Japan). The abundance of each zooplankton species/taxon was calculated by dividing its quantity by the filtered seawater volume, which was determined by two electronic flow meters equipped on MultiNet. Zooplankton abundance data are presented in ind/m^3^.

Zooplankton biovolumes were measured using the ZooScan Integrated System (HYDROPTIC, France), which can provide measures of zooplankton body size in terms of area (mm^2^) or volume (mm^3^) for each organism^25,61^. To ensure the accuracy of scanning and subsequent data, the preserved sample was first filtered through a 2-mm mesh size zooplankton sorting sieve and divided into two subsamples before using ZooScan for scanning measurement. The first subsample was all scanned and counted. The second subsample was split into 16–128 aliquots containing about 2000 objects with a Motoda box splitter. Each zooplankton sample was scanned in a 15 × 24 cm ZooScan scanning cell and digitized at a resolution of 4800 dpi. The zooplankton biovolume (displacement volume, DV) was calculated using the following formula:

$$ {\text{DV}} = ({4/3}) \times \pi \times ({\text{major/2}}) \times ({\text{minor/2}})^{{2}} , $$

where the major and minor axes (mm) of each object were provided by ZooScan. According to the ICES (International Council for the Exploration of the Sea) zooplankton methodology manual, the conversion factor between DV and dry mass (DM) is 160 mg DM/cm^3^ DV. The conversion factors for carbon and DM of different zooplankton taxa are as follows: 0.05 (Cnidaria, Ctenophora), 0.10 (tunicates), 0.27 (pelagic ostracods, planktonic pteropods, Heteropoda), 0.35 (Polychaeta), 0.40 (pelagic larvae, Amphipoda, Chaetognatha, Isopoda), 0.45 (Euphausiacea, Decapoda, Mysidacea), 0.50 (Copepoda, Cladocera)^62^. Zooplankton carbon biomass (CB) data are presented in mg C/m^3^.

The active carbon flux (CF) of the zooplankton community was calculated using the following formula^64^:

$$ {\text{R}} = {\text{RO}} \times 0.{97} \times ({12/22}.{4}), $$

$$ {\text{Fr}} = {\text{B}} \times {\text{R}} \times {\text{t}}, $$

$$ {\text{CF}} = {\text{Fr}} + {\text{Fe}}, $$

where RO is the respiratory metabolic rate, which is estimated with the empirical model of Ikeda^65^; 0.97 is the respiration quotient, which is the molar ratio of carbon dioxide produced to oxygen consumed; 12/22.4 is the mass (12 g) of carbon in 1 mol (22.4 L) of CO_2_; B (mg C/m^2^) is the diel-migrating zooplankton biomass, calculated as the difference between daytime and nighttime; R is the hourly carbon metabolism estimated from the body weight at the mean temperature of the 200–300-m layer; t is the average number of daylight hours (12 h in this study); Fr and Fe are the carbon respiration flux and the excretory carbon flux of the migrant zooplankton, respectively. Fe was calculated as 31% of Fr^66^. Zooplankton CF data are presented in mg C/(m^2^·d).

### Zooplankton community data analysis

The diversity and structure of the zooplankton community in the Caroline Seamount area were calculated and evaluated with the Shannon–Wiener index (*H'*), dominance index (*Y*), and Sørensen’s coefficient of similarity (*SCS*). The formulas of these indices are as follows:

$$ H\prime = - {\text{SUM}}({\text{P}}_{{\text{i}}} \times {\text{ln}}({\text{P}}_{{\text{i}}} )), $$

$$ Y = {\text{P}}_{{\text{i}}} \times {\text{f}}_{{\text{i}}} , $$

$$ SCS = {\text{2C/}}({\text{A}} + {\text{B}}) \times {1}00\% , $$

where Pi is the ratio of the abundance of species “i” to the total abundance of all zooplankton species, f_i_ is the occurrence frequency of species “i” in all samples, A and B are the number of zooplankton species/taxa collected at different sampling times, and C is the number of common zooplankton species/taxa collected at the two sampling times.

When its *Y* is higher than 0.02, the zooplankton species is considered a dominant species. For each dominant zooplankton species, the weighted mean depth (WMD) was calculated using the formula WMD = ∑ (n_i_ × d_i_ × z_i_)/∑ (n_i_ × z_i_), where d_i_ is the midpoint of each sampling layer, n_i_ is the abundance of species “i” at the d_i_ sampling layer, and z_i_ is the thickness of the d_i_ sampling layer^67^.

The contour figures of ST, SS, CHL, and DO for the five sampling times at station B3 were based on Kriging interpolation and generated with Golden Software Surfer 14. Pearson correlation analyses of zooplankton abundances and environmental factors (ST, SS, CHL, and DO) were statistically evaluated with IBM SPSS Statistics V20. A principal components analysis (PCA) was carried out using CANOCO for Windows V5.0 to analyse the impact of the environmental factors on the zooplankton community. To estimate which set of environmental factors best explained the zooplankton community structure, the BIO-ENV procedure was conducted with PRIMER V6.0 software.

## Supplementary Information

Supplementary Information.

## Data Availability

Datasets for this research are included in this paper and its supplementary information files.
